# A Case of Induction Chemoimmunotherapy and Sleeve Lobectomy to Avoid Pneumonectomy for Central Squamous Cell Lung Cancer

**DOI:** 10.70352/scrj.cr.24-0069

**Published:** 2025-02-22

**Authors:** Ayaka Asakawa, Ryota Ishizawa, Yukitaka Sato, Yuya Ishikawa, Ryo Wakejima, Hironori Ishibashi, Kenichi Okubo

**Affiliations:** Department of Thoracic Surgery, Graduate School of Medical and Dental Sciences, Institute of Science Tokyo, Tokyo, Japan

**Keywords:** neoadjuvant chemoimmunotherapy, lung cancer, sleeve lobectomy

## Abstract

**INTRODUCTION:**

Recently, neoadjuvant immunotherapy plus chemotherapy has been provided for patients with stage II-III resectable lung cancer. We report a case in which a pneumonectomy was avoided by administrating neoadjuvant chemoimmunotherapy.

**CASE PRESENTATION:**

An 81-year-old man presented with a cough. Examination showed squamous cell lung cancer in the right lower lobe extending to the central side of the upper lobe, which would have required a pneumonectomy for complete resection. Neoadjuvant chemoimmunotherapy was administered to reduce the extent of pulmonary resection due to the patient’s advanced age and impaired pulmonary function. Post-treatment examination showed tumor size reduction, and bronchoscopy showed disappearance of right upper bronchial erythema and persistent erythema of the bronchus intermedius. A sleeve right lower lobectomy was performed. Histopathological findings revealed complete resection of the cancerous lesion and a major pathological response.

**CONCLUSIONS:**

Sleeve lobectomy after preoperative chemoimmunotherapy for an elder patient with low pulmonary function was safe and efficient.

## INTRODUCTION

Induction therapy is used to treat locally advanced lung cancer. Recently, neoadjuvant immunotherapy plus chemotherapy has been provided for patients with stage II-III resectable lung cancer. Nivolumab, an immune checkpoint inhibitor (ICI) used for preoperative treatment of primary lung cancer, was reported in the CheckMate 816 study, showing significantly prolonged event-free survival (EFS) and greater pathological complete response (pCR) or major pathological response in patients treated with nivolumab and chemotherapy.^1)^ Herein, we report a case in which the administration of nivolumab plus chemotherapy prevented the need for pneumonectomy.

## CASE PRESENTATION

**Fig. 1:**
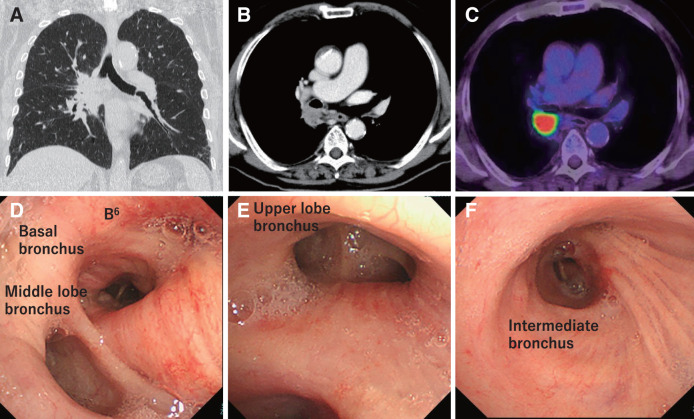
Imaging scan of the patient before chemoimmunotherapy. (**A, B**) Computed tomography scan showing a 51-mm mass in the right lower lobe, which extended to the dorsal side of the right upper lobe and hilar lymph node. (**C**): PET-CT showed tumor maximum standardized uptake value (SUVmax) was 13.3. (**D–F**) Bronchoscopy showing the occlusion of B6 and erythema of the bronchial mucosa on the dorsal surface of the upper lobe bronchus, intermediate bronchus, and lower lobe bronchus. PET-CT, positron emission tomography-computed tomography

An 81-year-old man complained of a cough. Blood examination showed mild chronic kidney disease, and a respiratory function test indicated the forced expiratory volume in 1 second (FEV1) of 1.60 L and the forced expiratory volume in 1 second divided by forced vital capacity (FEV1/FVC) of 59.70%. An imaging examination showed a 51 mm mass in the right lower lobe that infiltrated the right upper lobe and #11 hilar lymph node. Positron emission tomography-computed tomography (PET-CT) revealed no distant metastases. Bronchoscopy showed occlusion of B6 and erythema of the bronchial mucosa on the dorsal surface of the upper, intermediate, and lower lobe bronchi (**Fig. 1**), and transbronchial lung biopsy revealed squamous cell lung cancer. The preoperative stage of lung cancer was cT3N1M0, cStage IIIA. PD-L1 expression in the tumor was 30%–40% based on immunohistochemistry (28-8 pharmDx assay (Dako)). A right pneumonectomy was needed to extract all cancerous lesions. Considering low FEV1, FEV1/FVC, and the age of the patient, it was concluded that pneumonectomy should be avoided. Therefore, preoperative treatment was administered to reduce the extent of lung resection. The regimen included paclitaxel (200 mg/m^2^) plus carboplatin (5 areas under the blood concentration–time curve) and nivolumab (360 mg/body), determined based on advanced age and renal function, in accordance with the CheckMate 816 study.

**Fig. 2:**
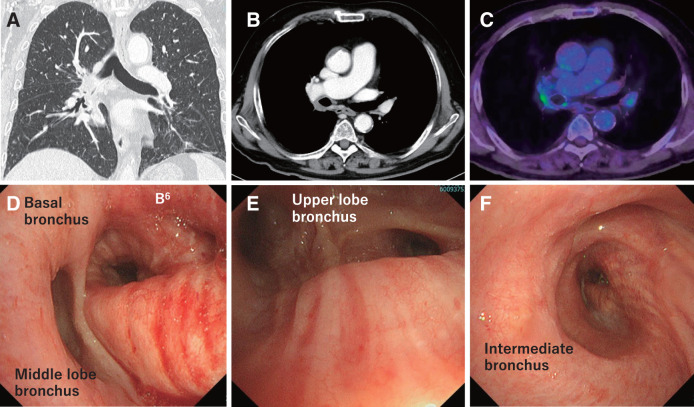
Imaging scan of the patient after chemoimmunotherapy. (**A, B**) Computed tomography scan showing that the tumor reduced size to 18 mm. (**C**) PET-CT showed tumor SUVmax was 3.9, decreasing from pre-treatment. (**D–F**): Bronchoscopy showing the erythema of the bronchial mucosa on the intermediate bronchus and the lower lobar bronchus. PET-CT, positron emission tomography-computed tomography; SUV, standardized uptake value

**Fig. 3:**
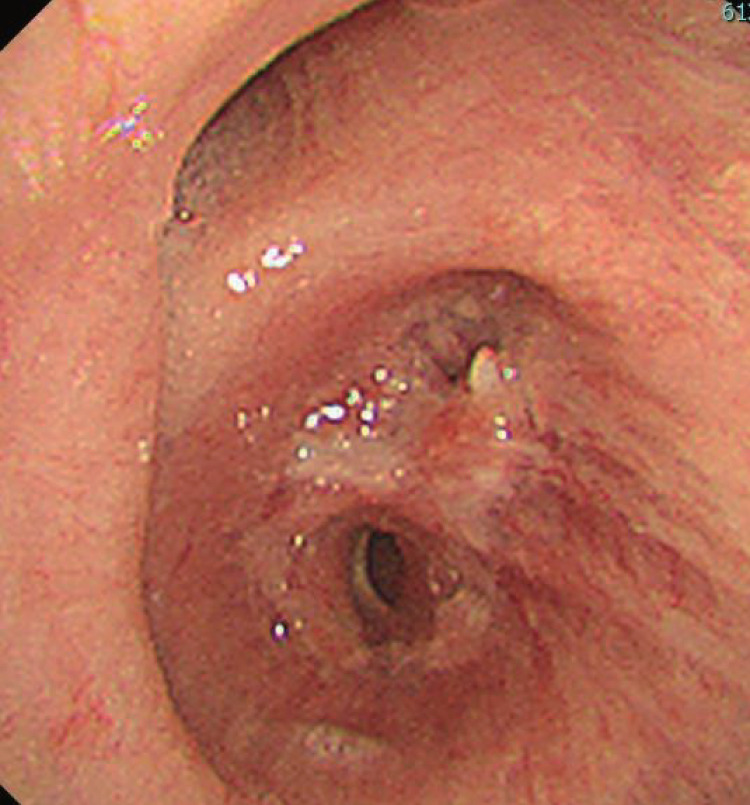
Bronchoscopy performed 1 month after surgery showing no signs of stenosis or anemic blood flow.

During the treatment, the patient experienced dizziness, which recovered quickly with intravenous infusion treatment, and finally completed three courses of the regimen. After neoadjuvant therapy, the respiratory function test showed FEV1 of 1.62L and FEV1/FVC of 52.59%, which had not improved. Post-treatment imaging showed that the tumor had reduced to 18 mm in size, and bronchoscopy revealed the disappearance of the right upper bronchial erythema, patency of B6, and remaining erythema of the bronchial mucosa on the intermediate and lower lobar bronchus (**Fig. 2**). PET-CT showed a decrease in fluorodeoxyglucose uptake in the tumor (13.3 to 3.9) and no signs of distant metastasis. The post-treatment stage was yc-T1bN0M0, Stage IA2, and surgery was performed as planned. There was no evidence of cancer invasion of the right middle lobe and the middle lobar bronchus, then preservation of the middle lobe was indicated. Additionally, if the intermediate bronchus margin was positive for cancer, pneumonectomy or complex reconstruction would be required. However, this was not indicated for this patient due to age and low pulmonary function. Therefore, the extent of the lung resection was planned to be a sleeve resection of the lower lobe. A right posterolateral thoracotomy was performed through the fourth intercostal space. Mild adhesions were observed on the dorsal side of the right lower lobe. The inferior lobar branch of the pulmonary artery and inferior pulmonary vein were divided. The middle lobar bronchus was transected at the bifurcation between B4 and B5. The intermediate bronchus was transected at 1 ring on the peripheral side of the right second carina. The pericardium underneath the hilum was incised as tension-reducing maneuvers. After extraction of the right lower lobe, the intermediate and middle lobar bronchi were anastomosed with interrupted sutures using 4-0 PDS II (Ethicon Inc., Johnson & Johnson Company, New Brunswick, NJ, USA), with a telescoping bronchial anastomosis technique. The anastomotic site was covered with an intercostal muscle flap. The postoperative course was uneventful, and the patient was discharged without the need for home oxygen therapy. A bronchoscopy performed 1 month after surgery showed no signs of stenosis or anemic blood flow (**Fig. 3**). Six months after surgery, no metastasis or recurrence was observed.

Histopathological findings showed a 4-mm residual squamous cell carcinoma at the peripheral side of the B6 bronchus, fibrosis and hyalinization of the intermediate bronchus with negative bronchial margins, and no lymph node metastasis. This indicates a major pathological response (residual viable tumor: 7.8%).

## DISCUSSION

In this case, preoperative treatment of an elderly patient with low pulmonary function prevented pneumonectomy and preserved pulmonary function. There have been reports of preoperative chemoimmunotherapy resulting in downstaging and enabling surgery to be performed,^2,3)^ achieving the same effect. Chemoradiotherapy, the conventional preoperative treatment for locally advanced primary lung cancer, is typically determined by age and performance status (PS) and is generally not recommended for elderly patients.^4,5)^ Moreover, the effects of radiotherapy can make surgery more challenging due to adhesions and fibrosis.^6)^ In this case, the patient’s age and PS were not indications for chemoradiotherapy; therefore, he was treated with neoadjuvant immunochemotherapy, which did not result in noticeable side effects. Only loose adhesions were observed during the present operation, and no impact was felt during the dissection of the vascular sheath or other manipulations, unlike after chemoradiotherapy status. However, lobectomy after chemoimmunotherapy is reportedly more challenging than standard lobectomy.^7)^ In addition, a potential risk of ICI treatment is immune-related adverse events, which occurs during and after ICI treatment. For the patients treated with ICI, careful follow-up is needed.

Recently, a Japanese cohort showed the preoperative benefits of CheckMate 816.^8)^ In this study, similar to a previous study, there was prolonged EFS and a higher rate of achievement of pCR in the nivolumab group. This cohort included patients who underwent lobectomy or pneumonectomy, but not patients who underwent sleeve resection in the chemoimmunotherapy group. To the best of our knowledge, this is the first report of sleeve resection using the CheckMate 816 regimen in Japan, in which sleeve lobectomy following chemoimmunotherapy was performed safely and effectively. To assess efficacy and safety in more detail, additional cases need to be accumulated.

## CONCLUSIONS

Preoperative chemoimmunotherapy prevents pneumonectomy in an elderly patient with low pulmonary function. Although sleeve resection after chemoimmunotherapy has rarely been reported, it was performed safely and efficiently.

## ACKNOWLEDGMENTS

We would like to thank Editage (www.editage.com) for English language editing.

## DECLARATIONS

### Funding

Not applicable.

### Authors’ contributions

AA and KO conceived the idea of this report.

AA described the original draft.

KO supervised the writing of the manuscript.

AA, RI, and KO performed the surgery.

AA, RI, YS, YI, RW, HI, and KO performed perioperative management on the patient.

All authors have read and approved the final version of the manuscript.

### Availability of data and materials

Not applicable.

### Ethics approval and consent to participate

All procedures followed were in accordance with the ethical standards of the responsible committee on human experimentation (institutional and national) and the Helsinki Declaration of 1964 and later versions. Ethical approval was not applicable as this was a case report.

### Consent for publication

The patient consented to reporting details of this case in a scientific publication.

### Competing interests

The authors declare that they have no competing interests.
